# Challenges in the diagnosis and management of IBD: a sub-Saharan African perspective

**DOI:** 10.1177/17562848231184986

**Published:** 2023-07-12

**Authors:** Gill Watermeyer, Leolin Katsidzira, Bright Nsokolo, Olusegun Isaac Alatise, Babatunde M. Duduyemi, Chris Kassianides, Phoebe Hodges

**Affiliations:** University of Cape Town and Groote Schuur Hospital, Anzio Road, Observatory, Cape Town 7945, South Africa; Internal Medicine Unit, Faculty of Medicine and Health Sciences, University of Zimbabwe, Harare, Zimbabwe; School of Medicine and Clinical Sciences, Levy Mwanawasa Medical University, Lusaka, Zambia; Division of Gastrointestinal/Surgical Oncology, Department of Surgery, Obafemi Awolowo University/Teaching Hospitals Complex, Ile-Ife, Osun, Nigeria; Department of Pathology, College of Medicine and Allied Health Sciences/Teaching Hospitals Complex Highest University of Sierra Leone, Freetown, Sierra Leone; Department of Medicine, Faculty of Health Sciences, University of Cape Town, Rondebosch, Western Cape, South Africa; Barts and the London School of Medicine and Dentistry, Blizard Institute, Queen Mary University of London, London, UK

**Keywords:** Crohn’s disease, diagnosis, endoscopy, histopathology, inflammatory bowel disease, radiology, sub-Saharan Africa, therapy, ulcerative colitis

## Abstract

With the exception of South Africa, inflammatory bowel disease (IBD) has long been considered uncommon in sub-Saharan Africa (SSA) with a dearth of peer-reviewed publications from the subcontinent. This most likely reflects underreporting as some cases may be missed due to the high burden of infectious diseases which may closely mimic IBD. In addition, many countries in SSA have limited endoscopic capacity, inadequate access to diagnostic imaging and a notable scarcity of histopathologists, radiologists and gastroenterologists. Beyond these obstacles, which significantly impact patient care, there are many other challenges in SSA, particularly the unavailability of key IBD therapies. In this review, we discuss barriers in diagnosing and managing IBD in SSA, as well as some of the initiatives currently in place to address these short comings.

## Introduction

The term inflammatory bowel disease (IBD) refers largely to two conditions, Crohn’s disease (CD) and ulcerative colitis (UC); chronic inflammatory disorders of the gastrointestinal tract for which there is currently no cure.^1^ Historically, IBD has been considered a condition of high-income countries, notably those in North America and Europe. Recently there has also been an increase in cases of newly diagnosed IBD in North Africa, Asia and South America; areas where IBD was previously considered uncommon. This observation appears to parallel the increased industrialization of these regions.^2^

In contrast, outside of South Africa, IBD has long been considered rare in SSA. The recent Global Burden of Disease study reported age-standardized prevalence rates in SSA ranging between 9.9 and 11.2 per 100,000 population.^3^ These rates are in stark contrast with traditional high-incidence countries such as those of North America, where a prevalence of 442 per 100,000 people was reported for 2017.^3^

Two systematic reviews published in 2020 identified fewer than 250 published cases of IBD in SSA, after excluding those from South Africa.^4,5^

This scarcity of cases may represent underreporting, a lack of awareness of IBD in the subcontinent leading to misdiagnosis, and less exposure to the environmental risk factors described in high-income populations.^6^ It is also likely that many cases of IBD in SSA are missed due to limitations in diagnostic and clinical capacity. It is unclear whether the perceived rarity of IBD is due to lower levels of susceptibility or is merely a reflection of lack of capacity to identify cases.^4–6^ It is instructive that although immigrants from SSA had a much lower risk of IBD than non-immigrant Canadians, the risk in their children was similar to that of non-immigrants, increasing dramatically within a generation.^7^ This implicates the predominant role of early childhood environmental factors in IBD, and it is reasonable to assume that these factors are increasingly present in selected settings in SSA. Thus, it is judicious to assume that the incidence of IBD may be increasing in SSA, particularly in the younger cohorts.

One of the main challenges in the management of IBD in SSA is making a timely and accurate diagnosis, as this requires a combination of clinical history, laboratory findings, imaging, endoscopy and histopathology. Unfortunately, these different facets of medicine are inadequate in SSA, albeit to varying extents, contributing to a delay in the diagnosis of IBD. Often IBD is mistaken for an infectious enteritis, given the high burden of infectious mimics.^4–6^Although acute bacterial and viral infections can cause a colitis, these are seldom confused with IBD, given the abrupt onset of symptoms, and lack of chronicity on intestinal biopsies. However, there are several chronic infections that can resemble UC and CD both clinically and endoscopically.^8–20^

**Figure 1.**
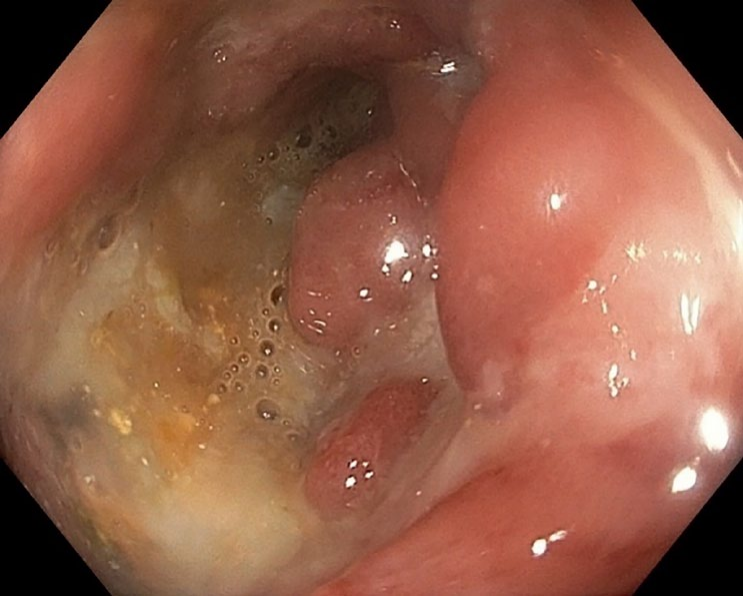
Deep ulceration and exudate in the caecum of a patient with amoebic colitis.

**Figure 2.**
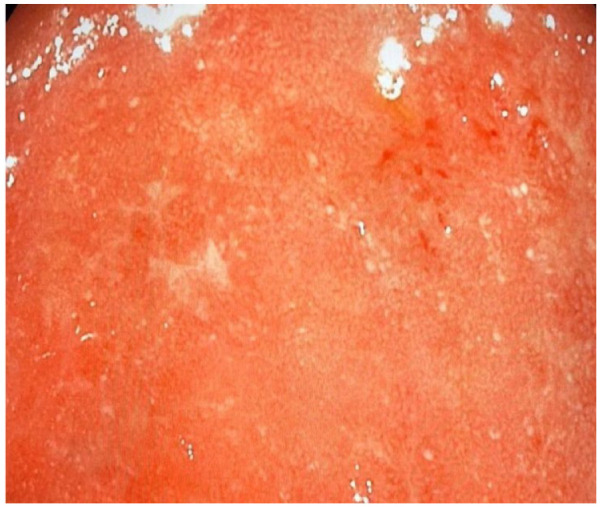
Extensive inflammation in the sigmoid colon of a patient with amoebic colitis.

These include *Entamoeba histolytica* which causes amoebic colitis.^8–10^*E. histolytica* can present with a wide range of clinical scenarios including asymptomatic colonization, self-limited diarrhoea, colitis and fulminant colitis.^8–10^ Endoscopic features include discrete ulcers with overlying exudate and normal intervening mucosa typically in the caecum or rectosigmoid (Figure 1), which may mimic Crohn’s colitis.^10^ However, in some patients, the endoscopic features may be indistinguishable from UC (Figure 2). The diagnosis of amoebic colitis is made on stool microscopy demonstrating cysts and trophozoites with evidence of haemophagocytosis, or on intestinal biopsies showing flask shaped ulcers and trophozoites.^8–10^ Amoebic serology or stool antigen testing may be of value in cases where stool microscopy and histology are unhelpful.^8–10^ Polymerase chain reaction (PCR) is considered the gold standard for the diagnosis of amoebiasis but widespread use in SSA is limited by cost and availability.^8–10^

Intestinal schistosomiasis due to *Schistosoma mansoni* is another common endemic infection which can mimic IBD in SSA.^9,11,12^ Typical symptoms include chronic or intermittent abdominal pain, poor appetite and diarrhoea. At endoscopy, the most common sites are the sigmoid colon and rectum, frequently with intestinal polyps. Rarely, strictures may develop. The diagnosis can be made on stool microscopy using the Kato-Katz method or on intestinal biopsies through identification of ova with a characteristic lateral spine. Granulomas are also typically present on histology.^11^ Serological testing, while useful for travellers from low-incidence regions, is of little value in SSA as most individuals have been exposed and are positive for antibodies. PCR, while useful, is not widely available.

Infection of the gastrointestinal tract with *Strongyloides stercoralis*, which is endemic in many countries in Africa, may also resemble IBD.^13,14^ Most infected persons will be asymptomatic or have mild nonspecific gastrointestinal (GI) such as watery diarrhoea, constipation, anorexia or weight loss.^13,14^ However, endoscopic findings of strongyloidiasis can be confused with those of IBD and the inadvertent use of high-dose corticosteroids can lead to *S. stercoralis* hyperinfection which carries a very high mortality.^13,14^ The diagnosis of strongyloidiasis is typically made by the presence of larvae on stool microscopy or in gut biopsies.^13,14^ A high peripheral eosinophil count or eosinophilic infiltrates on histopathology specimens should increase suspicion for this infection.^13,14^ However, it should be recognized that IBD may also be associated with a peripheral eosinophilia, introducing additional complexities.^15^

**Figure 3.**
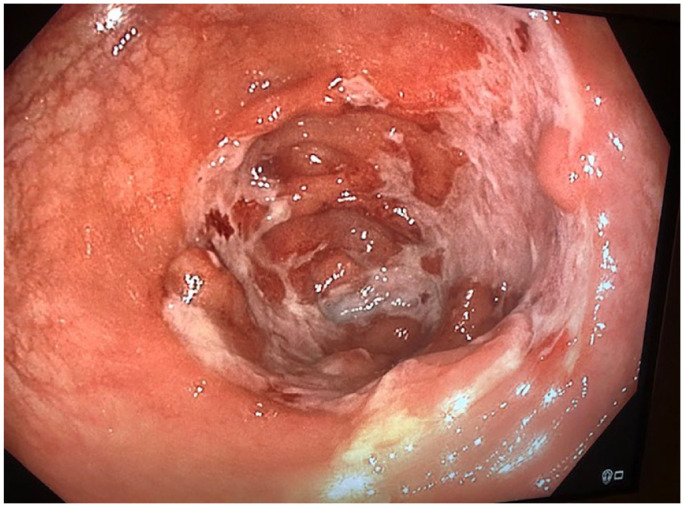
Circumferential ulceration and inflammation in the caecum in a patient with intestinal tuberculosis.

The main infectious mimic of CD in SSA is intestinal tuberculosis (ITB) caused by *Mycobacterium tuberculosis* (MTB); and differentiating these two granulomatous conditions is a major challenge in areas where tuberculosis (TB) is endemic, as they have overlapping clinical, endoscopic, radiographic and histological features.^16,17^ To make a definitive diagnosis of ITB one of the following must be present: caseating granulomas on biopsy, a positive TB culture, positive tissue GeneXpert MTB rifampicin (RIF) assay/TB PCR or the presence of acid-fast bacilli.^18^ Unfortunately, these diagnostic features are absent in the majority of cases of ITB and there is no perfect test to differentiate ITB from CD.^16,17^ Often, a combination of clinical, endoscopic, histopathological features, as well as findings on cross sectional imaging are required to make a diagnosis. A past history of TB or a positive TB contact will heighten suspicion of ITB, as will a chest X-ray or computerized tomography (CT) showing active or healed pulmonary TB.^16,17^ HIV positivity has been shown to favour a diagnosis of ITB over CD.^19^ Clinical features supporting CD include haematochezia, perianal disease, and extra-intestinal manifestations, while in ITB fever, night sweats, and ascites are common.^17,20^ Although most countries in SSA include Bacille Calmette-Guérin vaccination in their childhood immunization protocols which can result in a falsely positive tuberculin skin test (TST) later in life, a strongly positive TST or interferon-γ release assay will add additional support for a diagnosis of ITB.^20^ In the setting of HIV infection, urinary lipoarabinomannan (LAM) is a useful modality in the diagnosis of ITB.^21^ Certain findings on cross sectional imaging are helpful, with large or necrotic lymph nodes and ascites supporting ITB.^16,17,20^ Endoscopically, ITB manifests as transverse ulceration, pseudopolyps, or a patulous ileocaecal valve (Figure 3), whereas in CD longitudinal or aphthous ulcers, and a cobblestone appearance are typical.^16,17^ If non-caseating granulomas are present on histopathology, features favouring ITB include confluent, multiple, submucosal, or large granulomas. Additional findings supporting a diagnosis of ITB are bands of epithelioid histiocytes lining ulcers, and disproportionate submucosal inflammation.^16,17^ In cases of unresolved diagnostic uncertainty, an empiric trial of anti-TB therapy for 8–12 weeks is indicated.^16,17,20^

## Gastrointestinal endoscopy services in SSA

**Figure 4.**
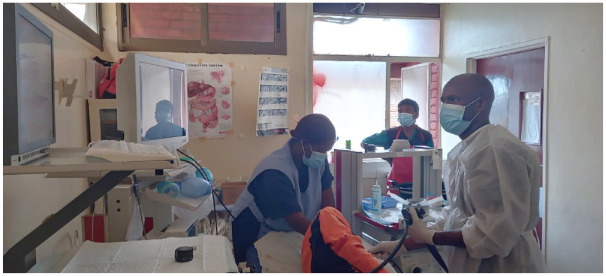
An endoscopic procedure being performed in the GI clinic at University Teaching Hospital, Lusaka, Zambia.

**Figure 5.**
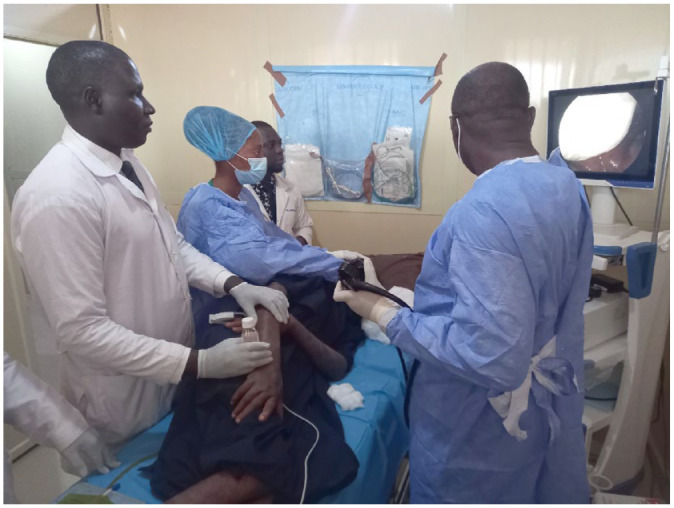
Endoscopy session at Obafemi Awolowo University Teaching Hospital Complex, Ile Ife, Nigeria.

Gastrointestinal endoscopy plays a central role in the diagnosis of IBD and although there are some centres in SSA that offer advanced endoscopy on par with high-income regions (Figures 4 and 5), access in many countries still presents a major challenge for multiple reasons, such as shortages of trained staff (both endoscopists and endoscopy nursing staff), lack of equipment, challenges in equipment maintenance, cost of the procedures and lack of judicious patient selection for endoscopic procedures among physicians.^6,22^

In a recent survey of digestive healthcare professionals from Ethiopia, Kenya, Malawi and Zambia, overall endoscopy capacity in the participating countries was approximately 0.12 endoscopists, 0.12 gastroscopes and 0.09 colonoscopes per 100,000 population.^23^ These values represent between 1% and 10 % of those reported from resource-rich countries. Perceived barriers to endoscopy services included a lack of equipment, trained endoscopists and costs. In this study, there was a median of one functioning colonoscope per participating facility, equating to 0.09 functioning colonoscopes per 100,000 population, when adjusted for non-response and additional facilities The overall capacity for lower GI procedures per year was 26.8 per 100,000 population, which compares to 3312.3 per 100,000 in the United States. Strikingly, there was approximately 1 endoscopist for every 400,000–2 million individuals in the participating countries.^23^ In another survey of physicians from West Africa, less than half had resources to perform Oesophago-Gastro-Duodenoscopy (OGD) and none could perform endoscopic retrograde cholangiopancreatography (ERCP).^24^ A general survey of 22 endoscopists practicing across 15 different SSA countries also highlighted the limited scope of endoscopy services and the need for additional training, particularly in therapeutic endoscopy, with only half of the participating countries reporting the existence of formal training centres.^24^ In this survey conducted by the European Society of Gastrointestinal Endoscopy, respondents rated IBD as the least prevalent of the diseases listed on the questionnaire, with the mean response being 2 on a scale of 1–5 where 1 = no prevalence and 5 = extremely prevalent.^25^ It is difficult to get a clear picture from endoscopic studies of the true prevalence of IBD due to the high burden of infectious diseases with similar appearances, which are often reported nonspecifically as ‘colitis’. For example, a Ghanaian study of patients presenting with haematochezia over an 11-year period found that nonspecific colitis was diagnosed in 34 out of 596 patients, equating to a rate of 5.7%.^26^ In contrast, the authors reported that UC was rarely diagnosed in this retrospective study.^24^ It is possible that the true rate of IBD was higher in this study, as some cases may have been described as ‘nonspecific colitis’. Similar rates of colitis are reported in other studies from SSA and these are summarized in Table 1.^27–35^ The highest reported rate was in the Zanzibar archipelago, Tanzania, where colitis was seen in 28.6% of colonoscopy procedures and was the commonest finding.^31^ It should be noted that most procedures in this study were performed by endoscopists from China, who may be more familiar with IBD and this may explain the higher rate of colitis.^31^ It is likely that most endoscopists in SSA require further training in recognizing IBD, and correlating these endoscopic findings with clinical and histopathological features.

**Table 1.** Rates of colitis seen in endoscopic studies in SSA.

Location	Rate of colitis	Time period	Inclusion criteria	Reference
Southern Nigeria	37/793 (4.7%)	January 2017–December 2019	Patients referred for colonoscopy with lower GI bleeding	Oluyemi *et al*.^27^
Zambia	27/526 (5.1%)	January 2008–December 2015	Unselected patients referred for colonoscopy	Kayamba *et al*.^28^
South-West Nigeria	12/250 (4.8%)	Not stated	Unselected patients referred for colonoscopy	Akere *et al*.^29^
Ghana	34/596 (5.7%)	January 1995–December 2000	Patients referred for colonoscopy for rectal bleeding	Dakubo *et al*.^26^
Cameroon	64/380 (16.8%)	January 2015–December 2015	Unselected patients referred for colonoscopy	Kamdem *et al*.2018^30^
Zanzibar Tanzania	128/448 (28.57%)	December 2013–October 2021	Unselected patients referred for colonoscopy	Qu and Gubi 2022^31^
Togo	13/180 (7.2%)	January–December 2018	Patients referred for colonoscopy with lower GI bleeding	Bagny *et al*.^32^
North-Central Nigeria	23/385 (5.9%)	March 2013–February 2020	Unselected patients referred for colonoscopy	Bojuwoye *et al*.^33^
Northern Nigeria	18.1%	January 2008–December 2017	Unselected patients referred for colonoscopy	Musa *et al*.^34^
Kenya	28.5%	January 1996–December 1997	Unselected patients referred for colonoscopy	Ogutu *et al*.^35^

SSA, sub-Saharan Africa.

This is supported by a study in which only one out of the four participating non-GI physicians from Nigeria, Senegal and the Gambia attending a short course on diagnosis and treatment of GI diseases reported receiving some training. All reported long waiting times for procedures and a lack of endoscopic resources.^24^

Barriers to endoscopy access in SSA countries also include lack of equipment and supporting infrastructure. In the aforementioned survey of endoscopists from 15 countries, 12 reported inadequate endoscopy services, with more than half of the countries reporting 0–20 endoscopy centres nationally. Automated disinfection of endoscopic equipment was not available in any of the countries surveyed. Infrastructure such as clean water and reliable electricity supplies were also notable barriers in running endoscopy units. Maintenance and repair of equipment was also a challenge, with only minor repairs and maintenance being carried out locally.^25^

Even in South Africa (SA), an upper middle-income country with one of the highest gross domestic products in SSA, major problems were identified in an audit of gastroenterology services in the Western Cape.^36^ Of the 4 tertiary hospitals, 5 regional hospitals and 10 district hospitals that were audited, only 2 were using automatic processing/disinfection. Although all centres offered diagnostic colonoscopy, the mean delay from consultation to procedure was 8 weeks. The major challenges identified were outdated and malfunctioning equipment, as well as the manual reprocessing of equipment, shortage of trained endoscopy nurses and a critical shortage of endoscopists, all resulting in delays in service delivery.^36^ In another study from South Africa, service disruptions caused by equipment breakages were reported, with an average of 3 gastroscope repairs and 1.25 colonoscope repairs required per hospital per year. Only 1 of the 11 institutions surveyed had an equipment service contract, and this was the only institution that did not report service interruptions due to breakages. Survey respondents cited lack of equipment, trained staff and equipment maintenance contracts as major challenges.^37^ Cost appears to be a major barrier to accessing colonoscopy for patients in SSA; an audit of colonoscopy practice in Nigeria cites the high cost of colonoscopy services and small scope of health insurance coverage, with out-of-pocket costs contributing to difficulties in accessing colonoscopy. In addition to this, there were limited public healthcare options, with only one of the nine centres a public institution, with most colonoscopy services delivered by private healthcare establishments.^38^

Patient and physician perceptions may also influence referral patterns for colonoscopy.^39^ In a study of health-seeking behaviour in patients with rectal bleeding in Nigeria over one-third of patients believed this was hereditary, only 39% knew that it could be a sign of cancer and only 39% had consulted a physician. Of those who had not consulted a physician, 40% had not considered rectal bleeding to be problematic, bleeding had resolved in 42%, 22% cited financial constraints and 18% sought herbal medicine.^36^ A total of 45 primary care physicians were also surveyed as part of this study, and although 73% saw patients with rectal bleeding at least once a month, the majority treated these patients conservatively and did not refer. Only three physicians reported immediate referral of their patients with rectal bleeding for specialist review. Over one-quarter of these physicians had never requested a colonoscopy for patients presenting with rectal bleeding.^39^

Recently, the COVID-19 pandemic affected delivery of gastrointestinal endoscopy services globally, and SSA was no exception.^40,41^ A survey that was undertaken in 2020 by the International Working Group of the European Society for Gastrointestinal Endoscopy and the World Endoscopy Organization on the impact of the COVID-19 pandemic on endoscopists in African countries found that 36% of respondents had reduced their endoscopy case volume by 75–100% and only 6% reported no reduction in case load.^41^ The vast majority also reported a change in their triaging practice, with most performing emergency and urgent procedures only, and 87% having postponed procedures indefinitely or for a period of at least 4 weeks. This was despite personal protective equipment including N95/FFP-2 masks being available in almost all participating centres.^41^

## Radiology services in SSA

Besides good clinical acumen, endoscopy, laboratory and pathology support, the diagnosis and management of IBD also requires the availability of basic and complex radiological services. Radiology is required during diagnosis, treatment monitoring and investigation of complications and extra-intestinal manifestations.^42,43^ While the availability of the whole gamut of radiological services is taken for granted in the Global North, this is frequently not the case in SSA. In many places, plain X-rays are often the only available resource and as such, judicious use is required for maximum benefit in IBD management.

At diagnosis, radiology is arguably more crucial with CD than UC, particularly for investigating and characterizing small bowel involvement.^42,43^ In recent times, magnetic resonance enterography has become the foremost investigation for this purpose.^42,43^ However, the density of magnetic resonance imaging (MRI) scanners in Africa is minuscule, averaging 0.8 scanners per million people, 40% of which are near-obsolete low-field systems and the MRI applications are heavily skewed towards musculoskeletal and neurological needs.^44^ This was also illustrated by a survey during a World Gastroenterology Organisation (WGO) training workshop in West Africa, which had participants from the Gambia, Nigeria and Senegal, of which only 7% had access to MRI.^24^ Lack of access to MRI also affects the assessment of extra-intestinal manifestations, particularly axial arthropathies and primary sclerosing cholangitis. Only 3.6% of participants had access to magnetic resonance cholangiopancreatography (MRCP) at this gastroenterology training workshop.^24^

In contrast, all participants in this survey had access to plain abdominal X-rays.^24^ Plain radiography is the most evenly distributed radiology resource in SSA, compared to computerized tomography (CT) and MRI, which are heavily skewed towards the urban centres, and the private, rather than public sector.^44–47^ Thus, the interpretation of basic plain abdominal X-rays is probably a much more critical skill for the gastroenterologist practising in SSA than would be the case elsewhere. The use of plain abdominal X-rays in IBD care in this setting range from a crude assessment of small bowel involvement in CD, evaluation of possible small bowel obstruction in complex CD, to assessing the large bowel, particularly in acutely unwell patients with UC or CD colitis, who may be at risk for toxic megacolon. Plain X-rays are also an important initial assessment of certain extra-intestinal manifestations, particularly the arthropathies, and for imaging of the bowel augmented by barium studies.

The other critical role of radiology at diagnosis, almost unique to SSA and Asia, is in the differentiation of the doppelgängers, CD and intestinal TB (ITB).^16,17^ Although of value if positive, CXR is usually normal in patients with ITB. CT or ultrasound scan (USS) of the abdomen are much more useful, with the presence of ascites, the characteristic lymph node and mesenteric involvement, and hepato-splenic lesions all favouring ITB.^16,17^ Unlike USS, CT is not as readily available, particularly within the public sector. In a review of 12 cases with IBD in Nigeria, only 1 had a CT scan over 3 years during the period under review.^48^ Although this may reflect the disease phenotype, it could also be an issue of availability. USS appears more widely available with 43% of participants at a WGO workshop having access to the modality.^24^ Beyond potentially helping distinguish ITB from CD, USS has increasingly become an acceptable method of assessing disease activity in both CD and UC.^49^ The wide availability of USS, including point-of-care devices, must be harnessed by the deliberate inclusion of USS training in gastroenterology curricula in the region.^50^

Finally, like other medical specialists, the number of radiologists in SSA remains very small, is unevenly distributed, and circumstances dictate that they practice as generalists, rather than subspecialize.^51^ This also impacts on the management of IBD in the region and requires attention to solid basic radiology grounding in gastroenterology training, exploring potential uses of teleradiology and networking across different countries in SSA.^50^

## Histopathology services in SSA

The histological examination of surgical specimens or endoscopic biopsies is a central element in establishing a diagnosis of IBD, and in differentiating UC and CD from other non-IBD-related conditions, in particular chronic infections. Accurate evaluation is dependent on good endoscopic and radiologic appraisal of the lesion; as such, detailed clinical, endoscopic and radiographic information should be made available to the pathologist.^52,53^

There are many challenges in the histopathological diagnosis of IBD in SSA, mostly human capital and infrastructure related.^22^ Unlike cancer, a definite histological confirmation of IBD is not possible. On the other hand, a diagnosis of either CD or UC cannot be entertained until infectious entero-colitis has been excluded on histopathology. The situation is complex in SSA where anatomical pathology capacity is limited. Many pathology laboratories do not have the infrastructure or technologies that are available in high-income countries. In addition, there is a dearth of qualified pathologists. On average, there is 1 pathologist for every million people in SSA compared with 1 per 15–20,000 people in the United Kingdom and the United States.^54^ These few pathologists are concentrated in a few countries such as South Africa and Nigeria, with some such as Liberia and Gambia having none in 2018.^54^

The quality of pathology reports has also been questioned, partly because most pathologists in SSA are generalists with very few specialists in GI diseases, potentially contributing to suboptimal analysis. In a study of findings from colonic biopsies performed during the workup of haematochezia in Ghana, nonspecific colitis was reported in 16.5%, while a definitive diagnosis of UC was made in only 7.3%.^26^ This may reflect inexperience in identifying IBD resulting in overreporting of ‘nonspecific’ colitis, highlighting the need for subspeciality training in the region.

The scarcity of pathologists in SSA can be in part attributed to insufficient trainees. Although pathology training programmes are offered in 25 countries, the output is inadequate for the region and this is compounded by the lack of harmonized curricula and accreditation.^55^ Moreover, there is inadequate exposure during some training programmes in histopathology in SSA. For example, the average number of all-organ specimens processed in 8 histopathology centres in Nigeria ranged from 2200 to 5500 per annum, while the average number of cases seen yearly per pathologist in these centres ranged between 420 and 786.^56^ These figures fall far short of the recommended numbers to ensure competency in high-income countries and is clearly suboptimal.^57^ On completion of pathology training there is the additional problem of retaining specialists in state institutions, as many opt to work in high-income countries or to move into the private sector. Factors that fuel this ‘brain drain’ include poor salaries, hostile working environments and limited opportunities for continuing education.^55^

## The management of IBD in SSA

Once a definitive diagnosis of IBD is made there are additional obstacles impacting patient care in SSA, in particular the unavailability of key therapies.^22^ While detailed information on medicine usage is lacking, treatment options appear to be limited for most countries in SSA, with substantial reliance on corticosteroids and aminosalicylates.^22,58–61^ Although, systemic corticosteroids remain the foundation of therapy for induction of remission in moderate-to severe UC and CD,^1^ they are often also used for long-term treatment in many countries as orthodox, guideline-based alternatives are often unavailable or are unaffordable. In general UC appears to be more common in SSA than CD, and as such, aminosalicylates are the most widely prescribed long-term therapy, especially sulphasalazine which is less costly than mesalazine products.^22^ Aminosalicylates are effective in mild-to-moderate UC for induction and maintenance of remission but have limited efficacy in CD.^1^ Despite the lack of evidence, many patients with CD in SSA are treated with sulphasalazine as it is often all that is available.^58,59^ The thiopurines, azathioprine and mercaptopurine, are effective treatments in the maintenance of remission in both CD and UC.^1^ Although little is known about the use of thiopurines in patients with IBD in SSA, evidence from reviews and reports from the rheumatological literature and a recent IBD survey indicate that azathioprine is available in some countries in the region.^22,62^

Methotrexate has proven efficacy in patients with CD but has not been shown to be effective in UCs.^1^ However, it is still widely used in patients with UC in SSA as the annual cost of methotrexate is lower than most other drugs, and it is available in many countries across the region.^22,62^

The availability of biological therapy in SSA is extremely low, and largely restricted to anti-TNFs which are formally approved in only a few countries^22,61^ Access to government-funded hospitals is best in South Africa, albeit only on a named-patient basis. Hopefully, the advent of less expensive infliximab and adalimumab biosimilars will improve access to biologic therapy in SSA, although currently, outside of South Africa, uptake is virtually non-existent.^20^ Other than costs, additional challenges affecting the availability of IBD medications in SSA include supply chain challenges, unfavourable manufacturing conditions, lack of effective regulations and circulation of fake and counterfeit compounds.^63^

For those patients fortunate enough to access anti-TNFs, there are additional challenges, notably the very high rates of latent TB and chronic hepatitis B viral infection in SSA with the potential risk of reactivation once exposed to anti-TNF’s.^22^ Outside of SA other therapies used in the treatment of IBD, such as cyclosporin, tacrolimus, ustekinumab, vedolizumab and tofacitinib, are generally not available in SSA.

Although considered a last resort in UC, surgical resection as a first-line therapy remains an option for some patients with isolated ileocaecal CD and may afford long-lasting surgical remission without medical therapy.^64^

## Measures underway to improve IBD diagnostic capacity and medicine availability in SSA

Many of the challenges facing the diagnosis and management of IBD in SSA reflect structural deficiencies in the healthcare systems, rooted in political and resource constraints. However, there are other, more specific barriers that are easier to address, such as educating healthcare workers about the diagnosis and management of IBD, facilitating patient advocacy and enhancing endoscopic and pathological capacity.^22^

Firstly, there is a need to raise awareness of IBD among clinicians, patients and policy-makers and several successful initiatives are on-going. IBD Africa, a non-profit organization founded in 2019, champions patient advocacy and education through several active programmes designed to improve patient care.^65^ A second non-profit organization, the Gastroenterology and Hepatology Society of Sub-Saharan Africa (GHASSA) is an association of gastroenterologists from 15 SSA countries, hosts weekly virtual teaching programmes on all aspects of gastroenterology and hepatology, including endoscopy, radiology, pathology and the management of IBD specifically.^66^ Both IBD Africa and GHASSA are working closely with pharmaceutical companies in the region to improve access to biologic therapies.^65,66^

Secondly, there is a need for structured training of healthcare workers in diagnosis and management of IBD, ideally within the context of gastroenterology fellowship training. This is an important unmet need in most countries in the region, with very few having ongoing established subspeciality programmes.

There is an urgent need to increase access to GI endoscopy in SSA through improving human capital and equipment in a sustainable way. Several approaches have been suggested or trialled to address the shortfall in capacity.^67–69^ One possibility is to establish a mobile colonoscopy service which would allow increased access to diagnostic endoscopy in SSA. This has been successfully used to screen a high-risk population for Lynch syndrome in a remote South African community with no access to endoscopy. The quality of this service was comparable to that of established endoscopy units.^70^

A number of initiatives have also been implemented to upscale training of endoscopists to address the human resource issues. Several training programmes have come about as a result of partnerships between endoscopy centres in high-income countries and centres in SSA.^67–69^ In general, these programmes entail supportive visits from qualified gastroenterologists to provide basic skills courses for those with little or no endoscopy experience or refresher/enhanced courses for those with more experience. The Master of Medicine in Gastroenterology programme established by the University of Zambia in recent years has this same aim: to provide comprehensive training for aspiring gastroenterologists locally, with endoscopy training being integrated into this programme.^71^

Another approach to endoscopic capacity building is that of training nonphysician clinicians to perform endoscopy. Clinical officers who are paramedical healthcare professionals with 4 years of formal training in Malawi, were trained to perform upper endoscopy. Over a period of 9 years, 1059 OGDs were carried out with no significant difference in failure or complication rates between procedures when compared to medical doctors.^72^ This approach may, however, not be advisable for colonoscopy which is widely accepted to be a more technically demanding procedure requiring a longer training period.

The other arm of IBD care, radiology, can be augmented in several ways in SSA, with Point-of-care ultrasound (POCUS) being the most promising. POCUS is widely used in day-to-day IBD care in many high-income countries.^73–75^ It is an increasingly accessible skill, and with newer technologies, POCUS can be performed using portable, pocket-sized mobile health devices such as smartphones and tablets. In addition, POCUS is becoming increasingly affordable.^73^ POCUS can fill the imaging gap where radiology services are absent, as is the case in many low-income SSA countries There are many examples of the successful use of POCUS in the region, mostly for obstetric, respiratory and cardiology indications.^74,75^ There is no data yet for IBD. Teleradiology is another potential modality to strengthen digital imaging in SSA.^76^ The benefits of teleradiology, have been well-described in the literature and teleradiology has been shown to be beneficial for both patients and physicians.^76^ A successful teleradiology programme will enable underserved communities to access investigations without the need to travel long distances and is a valuable tool to lessen workforce shortages in Low Middle Income Countries. Data on teleradiology in SSA are sparse but what little are available suggest that it is not yet employed to its full capacity.^76^

The need to improve pathology services in SSA has also long been recognized.

The establishment of the College of Pathology of East, Central and Southern Africa (COPECSA) is an important initiative in addressing these deficiencies.^77^ In partnership with various international organizations, COPECSA has successfully implemented programmes and projects in which more than 100 pathologists have been trained in various aspects of pathology and laboratory medicine. COPECSA also aims to facilitate the harmonization of pathology training and standardize the practice of pathology within the region.^77^ Apart from regional initiatives, individual countries are also introducing new postgraduate training programmes in pathology despite the challenges. For example, a postgraduate pathology training programme was launched in Zambia, in 2011. Four Zambian pathologists have already graduated with 10 more in training.^78^

As with radiology the development of telepathology has the potential to improve pathology services. In SSA telepathology has been used successfully for teaching as well as diagnosis.^79^ As an example, in Malawi, at the Kamuzu Central Hospital, high-resolution digital slides were transmitted to collaborating pathologists in the United States for diagnosis.^80^ A similar arrangement between the Department of Pathology at Mulago Hospital in Uganda and Fuerth Hospital in Germany has been reported.^81^

Following recent initiatives to raise awareness of IBD in SSA a number of case series and cohort studies have been published which have added to the numbers identified in the aforementioned systematic reviews, suggesting that the incidence of IBD in the subcontinent is likely on the increase.^27,31–34,82,83^

While comprehensive epidemiological data are lacking, there are many plausible environmental explanations for this observation, such as changes in diet from the traditional African diet rich in fibre, fruit and vegetables, to a more westernized diet high in sugar, saturated fat, emulsifiers and preservatives which appear to increase the risk of developing IBD.^6^ In addition, the widespread eradication of Helminth infections, Schistosomiasis and *Helicobacter pylori*, which may confer a protective effect, could have contributed to an increase in cases in SSA.^6,84^ Given that SSA may be one of the last regions on the globe to enter the early stages of the IBD epidemiological transition, the subcontinent offers the perfect opportunity to study the aetiopathogenesis of both CD and UC. Large scale epidemiological studies are required focussing on environmental risk factors, the microbiome and genetic susceptibility in SSA. Such research could provide invaluable insight into some of the many scientific uncertainties of IBD. The rapidly growing field of Artificial intelligence will also go a long way in increasing diagnostic capacity and supporting such initiatives.^85^

## Conclusion

With the exception of South Africa, IBD has long been considered uncommon in SSA, with few peer-reviewed publications from the subregion. This most likely reflects underreporting, however, some cases may be missed due to the high burden of infectious diseases which closely mimic IBD. In addition, many countries in SSA have limited endoscopic capacity, inadequate access to diagnostic imaging and a notable scarcity of histopathologists, radiologists and gastroenterologists. Beyond these obstacles which significantly impact patient care there are many other challenges in SSA, in particular the unavailability of key IBD therapies. There are a number of initiatives aimed at increasing the number of qualified gastroenterologists, endoscopists and histopathologists, as well as heightening awareness of IBD in SSA currently underway to address these shortcomings.

## Declarations

**Ethics approval and consent to participate:** Not applicable.

**Consent for publication:** Not applicable.

**Availability of data and materials:** Not applicable.
